# The Effect of AMF Suppression on Plant Species Composition in a Nutrient-Poor Dry Grassland

**DOI:** 10.1371/journal.pone.0080535

**Published:** 2013-11-12

**Authors:** Tomáš Dostálek, Hana Pánková, Zuzana Münzbergová, Jana Rydlová

**Affiliations:** 1 Institute of Botany, Academy of Sciences of the Czech Republic, Průhonice, Czech Republic; 2 Department of Botany, Faculty of Science, Charles University, Prague, Czech Republic; University of Saskatchewan, Canada

## Abstract

Arbuscular mycorrhizal fungi (AMF) are expected to be one of the key drivers determining the diversity of natural plant communities, especially in nutrient-poor and dry habitats. Several previous studies have explored the importance of AMF for the composition of plant communities in various types of habitats. Surprisingly, studies of the role of AMF in nutrient-poor dry grassland communities dominated by less mycotrophic plant species are still relatively rare. We present the results of a 3-year study in which a plant community in a species-rich dry grassland was subjected to the fungicide carbendazim to suppress AMF colonization. We tested the effect of the fungicide on the following parameters: the plant species composition; the number of plant species; the cover of the rare, highly mycorrhiza-dependent species *Aster amellus*; the cover of the dominant, less mycorrhiza-dependent species *Brachypodium pinnatum*; and the cover of graminoids and perennial forbs. In addition, we examined the mycorrhizal inoculation potential of the soil. We found that the suppression of AMF with fungicide resulted in substantial changes in plant species composition and significant decrease in species richness, the cover of *A. amellus* and the cover of perennial forbs. In contrast the species increasing their cover after fungicide application were graminoids—the C3 grasses *B. pinnatum* and *Bromus erectus* and the sedge *Carex flacca*. These species appear to be less mycorrhiza dependent. Moreover, due to their clonal growth and efficient nutrient usage, they are, most likely, better competitors than perennial forbs under fungicide application. Our results thus suggest that AMF are an essential part of the soil communities supporting a high diversity of plant species in species-rich dry grasslands in nutrient-poor habitats. The AMF are especially important for the maintenance of the populations of perennial forbs, many of which are rare and endangered in the area.

## Introduction

Arbuscular mycorrhizal fungi (AMF) can significantly influence the composition of grassland communities as well as ecosystem processes such as the uptake of nutrients by plants and the competitive relationships between plants in these communities [1-3]. At the individual level, root colonization by AMF results primarily in increased plant growth due to the improved acquisition of nutrients, especially phosphorus, by the plant [4,5]. The mutualistic relationship between AMF and plants usually occurs in soils with limited nutrient availability [5]. 

Although the effects of AMF on individual plant growth can be tested relatively easily in greenhouse experiments, the effects of AMF at the community level are much more difficult to test and depend on the specific species composition of the community, soil conditions and many other biotic and abiotic factors [6]. The effect of AMF on species diversity in natural plant communities can range from positive [1] over neutral [7] to negative [3]. The modification of arbuscular mycorrhizal symbioses can have significant consequences for the competitive relationships between different plant species. These relationships represent an important factor driving the diversity and composition of plant communities [8-11]. Hartnett and Wilson [12] have hypothesized that the direction in which mycorrhizal symbiosis will influence plant species diversity in the community depends on whether the dominant competitors are significantly more or less mycotrophic than their neighbors. If the dominant species are more mycotrophic, the presence of AMF tends to decrease species diversity by increasing the competitive ability of the dominant species [12,13], whereas the opposite occurs if the dominant species are less mycotrophic [14].

The most efficient way to test the effect of AMF on plant growth and diversity in field experiments is the suppression of AMF development in the soil using fungicides [15], with the fungicide benomyl the most prominent among the fungicides previously used for this purpose. Although the use of benomyl has certain limitations because it can change soil properties, e.g., nutrient concentration or pH, it has only limited side effects on plants and non-target organisms, e.g., [16,17]. To date many studies have been published on the effects of the suppression of AMF with fungicides on the plant species composition of a wide range of plant communities under the field conditions. Most of the studies have been performed in prairies dominated by C4 tallgrass species [3,12,18-20], but several studies have also been conducted in woodland and shrubland communities [13,21], subarctic forest community [22], temperate forest understory [23], lowland heaths [24], weed communities [25], boreal grassland [26], wet grassland [7], a serpentine site with annuals [27] and a calcareous thin-layered rendzina soil polluted with emissions of a phosphate fertilizer factory [28]. There is, however, only one study, conducted by Karanika et al. [15] in Greece, exploring the effect of suppression of AMF with fungicide on species diversity and composition in a nutrient-poor grassland dominated by less mycotrophic plant species. 

The lack of studies in nutrient-poor dry grasslands is surprising, as plants under these nutrient-poor dry conditions tend more to depend upon AMF than plants in habitats that are more nutrient rich and wetter [5,23]. In addition, dry grasslands contain many rare species, and the protection of such habitats is a major task in species conservation [29]. 

In this study, we present the results of a 3-year study in which a species-rich dry grassland community was treated with the fungicide carbendazim to suppress AMF development in the soil. We expected the fungicide to reduce AMF effectively [30]. Obligate mycotrophs may be less competitive if AMF are suppressed [26,31] and AMF associations may be important for seedling establishment [1,32]. We therefore hypothesized that the reduction of AMF would suppress the growth of highly mycorrhiza-dependent plant species and enhance the growth of less mycorrhiza-dependent dominants, thus causing plant species richness to decrease over time. To test this hypothesis, we compared the following variables in plots with and without fungicide application: the plant species composition; the number of plant species; the cover of the rare, highly mycorrhiza-dependent species *Aster amellus*; the cover of the dominant, less mycorrhiza-dependent grass *Brachypodium pinnatum*; the cover of graminoids; and the cover of perennial forbs. In addition, we examined the mycorrhizal inoculation potential in the soil, the nutrient concentration in the biomass of *B. pinnatum* and the soil chemical composition in the last year of the experiment.

## Materials and Methods

### Study area

Our study area is a species-rich calcareous dry grassland near Roudnice nad Labem (northern Bohemia, 50°30'2.627"N, 14°18'57.45"E), Czech Republic. Based on our previous studies, this site was selected to represent a typical locality of the region [33]. The grassland is situated on marl [34], and its vegetation belongs to the Bromion community [35,36]. No specific permits were required for the fieldwork described. 

Many localities in the region were managed in the past, and the distribution of plant species at the localities is strongly affected by past land use [33]. In addition, the diversity of the plant communities in the dry grasslands in the study is limited by the dispersal ability of the species and the overall availability of the species in the landscape [37-40].

### Fungicide application experiment

Twenty plots (1 x 1 m) were placed in a 30 x 10 m area on the dry grassland site. The plots were arranged in 10 blocks. Each block consisted of one plot with fungicide application and one control plot without fungicide application. The distance between the 2 plots within a block was 1 m. The blocks were organized in 2 parallel transects along the longer dimension of the study area. AM symbiosis was suppressed with the fungicide carbendazim (Karben Flo Stefes, Bayer CropScience, Frankfurt/Main, Germany). This fungicide contains the same active ingredient as the formerly used fungicide benomyl, which is not manufactured since 2001 [41]. Benomyl has been the fungicide utilized most widely to manipulate AMF communities in field experiments, although it is not specific to AMF and can also influence certain non-target organisms, such as the bacterial community, e.g., [12,13,16,21]. Benomyl (methyl-1-(butyl-carbamoyl)-2-benzimidazole) is enzymatically hydrolyzed to the active compound carbendazim (methyl 2-benzimidazolecarbamate, MBC) within a few hours after addition [42] and to 1-butyl isocyanate (BIC) [43,44]. A total of 100 ml of the fungicide carbendazim (Karben Flo Stefes) was diluted in 3 liters of distilled water and applied to the experimental plot in each block every 4 weeks throughout the growing season in 3 consecutive years (June to September in 2007 and March to September in 2008 and 2009). The control plot in each block obtained the same amount of water as the experimental plots.

Plant species composition was estimated using the new Braun-Blanquet cover scale [45] in an 0.5 x 0.5 m square in the middle of each plot. The rest of the plot was considered as a transitional area. Plant species composition was always recorded during the same period of the vegetation season (June 2007-2009). In further analyses we focused on different components of the plant community: the cover of the mycorrhiza-dependent species *A. amellus* (hexaploid cytotype) and the cover of the dominant, less mycorrhiza-dependent species *B. pinnatum* (whose mycorrhiza growth response in the target soil is one order of magnitude lower than that of *A. amellus*; A. Voříšková et al., unpublished data). Both of these species were present in all studied plots at the beginning of the experiment. In the study area, *Aster amellus* is represented by 2 cytotypes (diploid and hexaploid, [46]). Although the diploid cytotype used in most of our previous studies were proved to be highly mycorrhiza dependent [47,48], the study site hosts a population of the hexaploid cytotype. Sudová et al. [49] demonstrated, in a greenhouse experiment, that the hexaploid cytotype may be less responsive to AMF than the diploid cytotype. The response of the hexaploid *A. amellus* cytotype to AMF under natural conditions is, however, unknown. The initial data on plant species composition were pre-treatment, i.e., recorded before the fungicide was applied for the first time in June 2007.

The mycorrhizal inoculation potential of the soil (MIP; the potential of AMF propagules present in the soil to establish mycorrhizal colonization in roots of the host plant) in each plot was estimated before fungicide application in June 2007 and then twice during the experiment in June 2008 and 2009. To estimate MIP, we used a standard bioassay approach with maize (a universal AMF host) as a host plant [50]. In June of each year, we carefully removed 10 x 10 x 10 cm cube of soil from the transitional area at the very edge of each experimental plot. Accordingly, the vegetation was not disturbed in an 0.5 x 0.5 m square in the middle of each plot, where the plant species composition was recorded. The soil from each cube was homogenized and diluted with γ-sterilized soil taken from the studied locality in a ratio of 1:100 (v:v). The prepared substrates were used to fill into 125 ml pots. One pre-germinated maize seed (*Zea mays* L. cv. TATO) was planted in each pot. Six replicates were used for each sample. The plants were placed in a temperature-controlled greenhouse for 6 weeks. At harvest, the roots were washed and stained with 0.05 % trypan blue in lactoglycerol [51]. MIP was estimated as the percentage of the root length of the host plant colonized by AMF. Colonization was assessed using a gridline-intersect method on 200 intersects per sample [52] under a dissecting microscope at 40× magnification. As the MIP results from 2008 and 2009 were very similar, we will present only the MIP data from 2009. Prior to fungicide application in 2007, the MIP values were extremely low, ranging to zero. For this reason, the MIP was not evaluated in 2007. These low values were most likely result of an unknown technical problem. However, we do not expect significant differences in the initial MIP between plots with and without fungicide treatment because there were no statistically significant differences in MIP among control plots without fungicide application at the end of the experiment in 2009 (F_9,50_ = 1.55; P = 0.29), indicating a low level of spatial variation in MIP over the site.

Furthermore, samples of the aboveground biomass of *B. pinnatum* were taken from all plots in June 2009. *B. pinnatum* is a dominant species at the locality, and the analysis of the phosphorus concentration in its biomass provides information on the effects of fungicide on phosphorus uptake by the plants. The samples were dried in an oven at 80 °C, the dried leaves were homogenized in a grinding mill and the phosphorus concentration was analyzed using the method of Ehrenberger and Gorbach [53]. Our aim was also to analyze the phosphorus concentration in *A. amellus*, but the amount of biomass available was insufficient for this analysis.

The samples collected for MIP in June 2009 were also used for soil chemical analyses. We analyzed the pH using deionized water and a 0.1 M solution of KCl as the extraction agents. Total N and C, organic C and carbonate content and the concentrations of extractable Ca^2+^, Mg^2+^, K^+^ and available P were evaluated using the methods described in Pánková et al. [47].

#### Data analyses

We used a redundancy analysis (RDA) to test the effects of fungicide application, year and their interaction on plant species composition in the studied plots. Plots were used as whole plots, and records from 3 years within each plot were used as split plots. To test the effect of the fungicide, whole plots were freely permuted within blocks, whereas split plots were not permuted. Time was used as a covariate in this test. To test the effect of year – whole plots were not permuted, while split-plots were permuted along a linear transect within whole plots. Fungicide was used as a covariate in this test. To test the fungicide × time interaction, whole plots were freely permuted, whereas split plots were permuted along a linear transect within whole plots. Both time and fungicide application were used as covariates in this test. The cover values were transferred to percentage data (mid-value of each cover class interval) and square-root transformed as recommended by Lepš and Šmilauer [54]. We only used data on species that were recorded at least 4 times in the vegetation data during the experiment. Multivariate analyses were performed using Canoco for Windows 4.5 [55].

To test the effects of fungicide application, year and their interaction on number of plant species within plots, we used a Generalized Linear Model (GLM) with a Poisson distribution. To test the effects of fungicide application, year and their interaction on the cover of rare *A. amellus* and on the cover of dominant *B. pinnatum*, the summed cover of all graminoids and all perennial forbs, we used a factorial ANOVA. In both the GLM and the factorial ANOVA, block was used as a covariate.

To assess the effect of graminoids on species richness, we used a linear regression to test the relationship between the change in the cover of graminoids and the change in species richness in plots with and without fungicide application.

Differences in MIP between plots with and without fungicide application at the end of the experiment were tested with a factorial ANOVA where block was used as the covariate and the percentage of the root length of the host plant colonized by AMF (square-root transformed to obtain a normal distribution) as the dependent variable.

To compare the differences in phosphorus concentration in the aboveground biomass of *B. pinnatum*, the pH_H20_ and pH_KCl_ and the concentrations of Mg, Ca, K, P, and N, C_tot_, C_(C03)2-_ and C_ox_ in the soil at the end of the experiment, we used a non-parametric Kruskal-Wallis test with fungicide application as an independent variable. The results for pH_KCl_ were very similar to those for pH_H20_. For this reason, we will only present the results for pH_H20_. 

Significant differences in the soil properties between plots with and without fungicide could indicate that the effect of fungicide may be not due to the suppression of AMF but to changes in soil chemistry. If we found significant differences in chemical properties between plots with and without fungicide application, we repeated the tests of the effect of fungicide on plant species composition, the number of plant species, the cover of *A. amellus*, the cover of *B. pinnatum*, the cover of graminoids and the cover of perennial forbs with the given chemical property as a covariate. Because data on soil chemistry were only collected during the last year of the experiment, we also tested the effect of fungicide on the dependent variables without any covariate and compared the results. The effect of the significant soil property on all the dependent variables with fungicide as a covariate and without any covariate was tested as well. The effect on plant species composition was tested using an RDA; the effect on the number of plant species was tested using a GLM with a Poisson distribution; and the cover of *A. amellus*, *B. pinnatum*, graminoids and perennial forbs was tested using a factorial ANOVA. Block was used as a covariate in these analyses to remove the effect of the variability among blocks.

## Results

**Figure 1:**
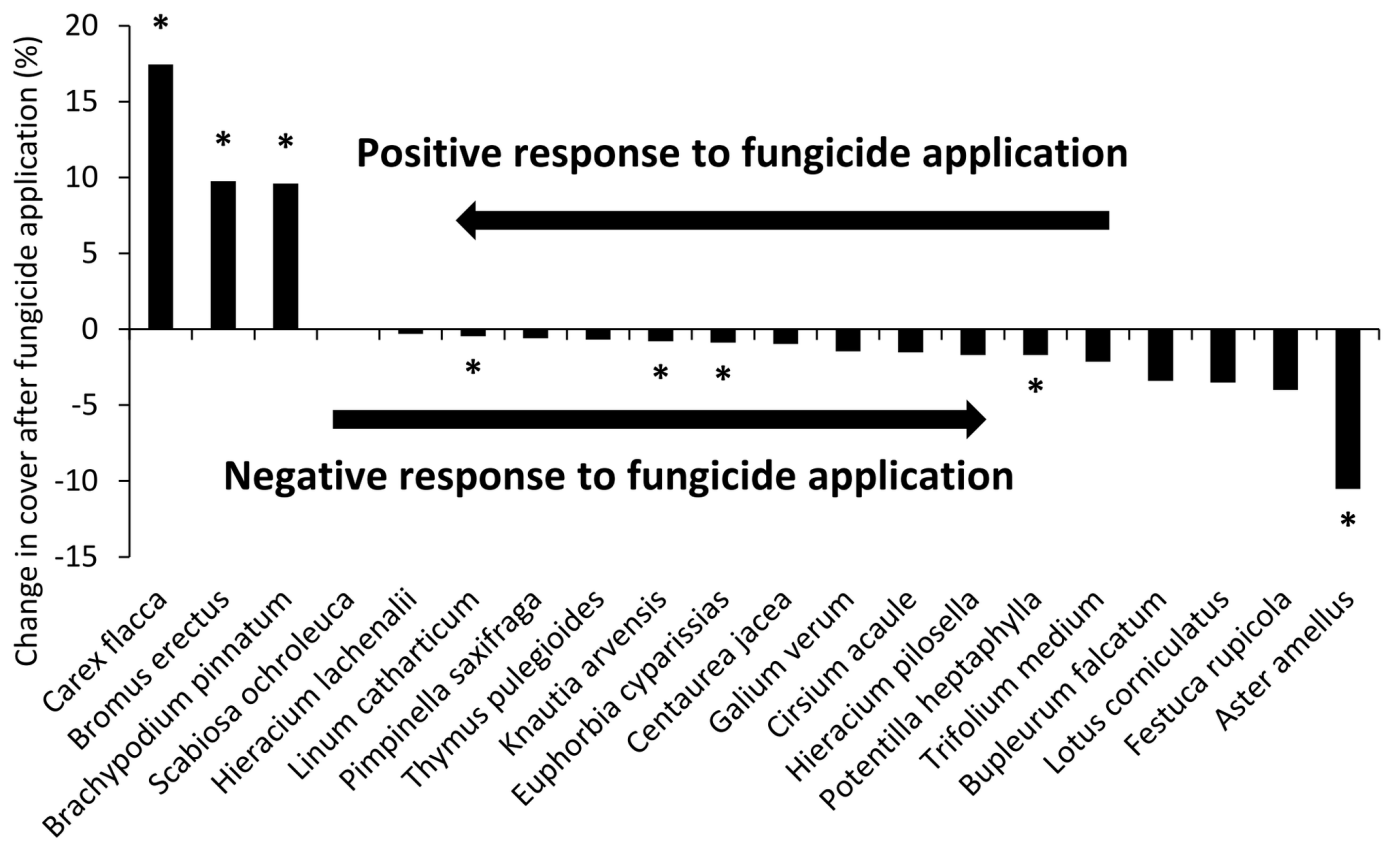
Species most positively and negatively affected by fungicide application. Graph shows change in the absolute cover of particular species after 3 years of fungicide application, averaged over 10 plots. Asterisks indicate significant (P ≤ 0.05) differences in species cover in the last year of the experiment between plots with and without fungicide application tested with a factorial ANOVA with block used as a covariate.

We found that fungicide application, year and their interaction all had significant effect on the plant species composition of the plots. The year explained the lowest proportion of the total variation in the data and the interaction of fungicide and year explained the highest proportion (Table 1). Only a few species showed a positive response to fungicide application - *Carex flacca*, *Bromus erectus* and *Brachypodium pinnatum*. A negative response was observed for most species. The species with the strongest statistically significant negative response were *Aster amellus*, *Potentilla heptaphylla*, *Euphorbia cyparissias*, *Knautia arvensis* and *Linum catharticum* (Figure 1).

**Table 1:** Effects of block, fungicide, year and interaction of fungicide and year on plant species composition, number of plant species, cover of *Aster amellus, Brachypodium pinnatum*, graminoids and perennial forbs.

	Resid. df	Plant species composition			Number of plant species			Cover of *A. amellus*			Cover of *B. pinnatum*			Cover of graminoids			Cover of perennial forbs		
		%	F	P	R^2^	F	P	R^2^	F	P	R^2^	F	P	R^2^	F	P	R^2^	F	P
Block	50	**5.9**	**3.6**	**0.036**	0.11	2.3	0.028	**0.25**	**2.3**	**0.026**	**0.26**	**2.5**	**0.022**	**0.18**	**2.6**	**0.016**	**0.21**	**3.2**	**0.004**
Fungicide	49	**8.5**	**5.2**	**0.008**	**0.25**	**48.1**	**<0.001**	**0.05**	**4.0**	**0.046**	**0.12**	**10.0**	**0.003**	**0.27**	**35.0**	**<0.001**	**0.19**	**26.3**	**0.003**
Year	48	**3.5**	**2.1**	**0.002**	**0.11**	**21.0**	**<0.001**	*0.10*	*8.2*	*0.095*	0.00	0.0	0.841	0.01	1.5	0.230	**0.14**	**18.9**	**<0.001**
Fungicide × year	47	**6.4**	**3.8**	**0.002**	**0.29**	**55.3**	**<0.001**	*0.06*	*5.2*	*0.061*	*0.05*	*3.9*	*0.053*	**0.18**	**3.4**	**<0.001**	**0.11**	**14.3**	**<0.001**

Significant values (P ≤ 0.05) are in bold. Marginally significant values (P ≤ 0.1) are in italics. Plant species composition was tested using a multivariate redundancy analysis, number of plant species using a GLM with a Poisson distribution and cover of *A. amellus*, *B. pinnatum*, graminoids and perennial forbs using a factorial ANOVA.

**Figure 2:**
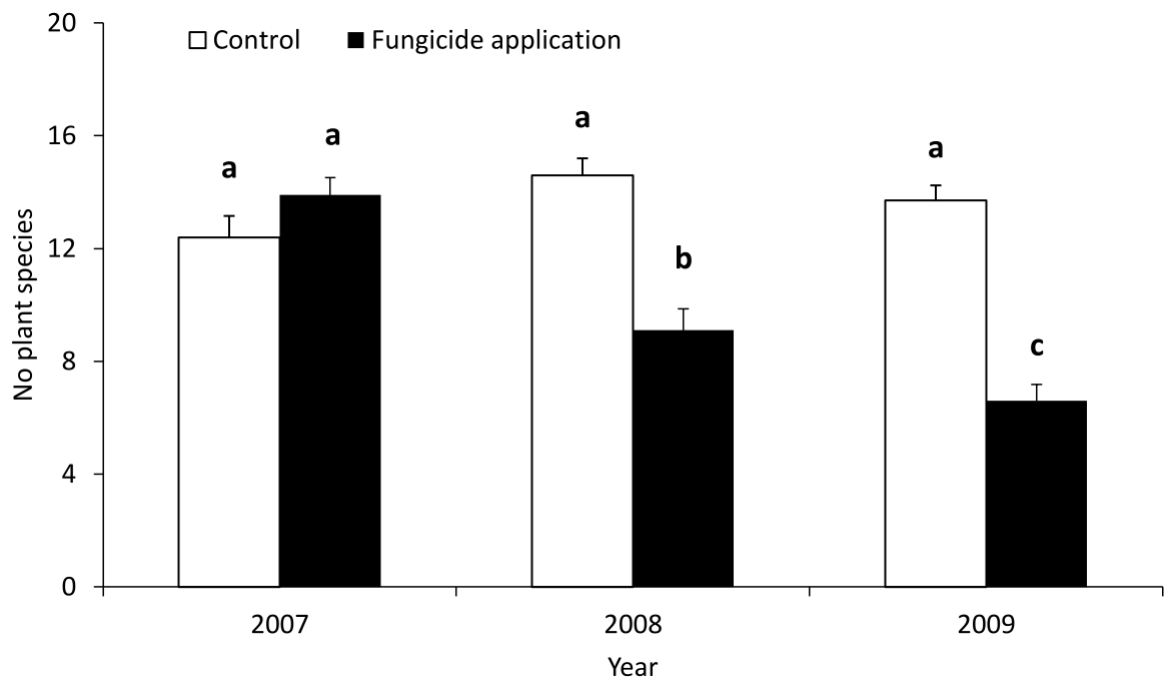
Number of plant species in plots with fungicide application and in control plots. The graph shows means and standard errors (n = 10). Columns marked by the same letter are not significantly different (P > 0.05). Tests were performed using a GLM with a Poisson distribution, with block as a covariate.

An overall negative response to fungicide application also resulted in a significant decrease in the number of species in the plots where fungicide was applied (Table 1). At the beginning of the experiment, an average of 14 species was recorded in each plot. In the last year of the experiment, we recorded, on average, only 7 species in the plots with fungicide application and 14 species in the control plots (Figure 2).

**Figure 3:**
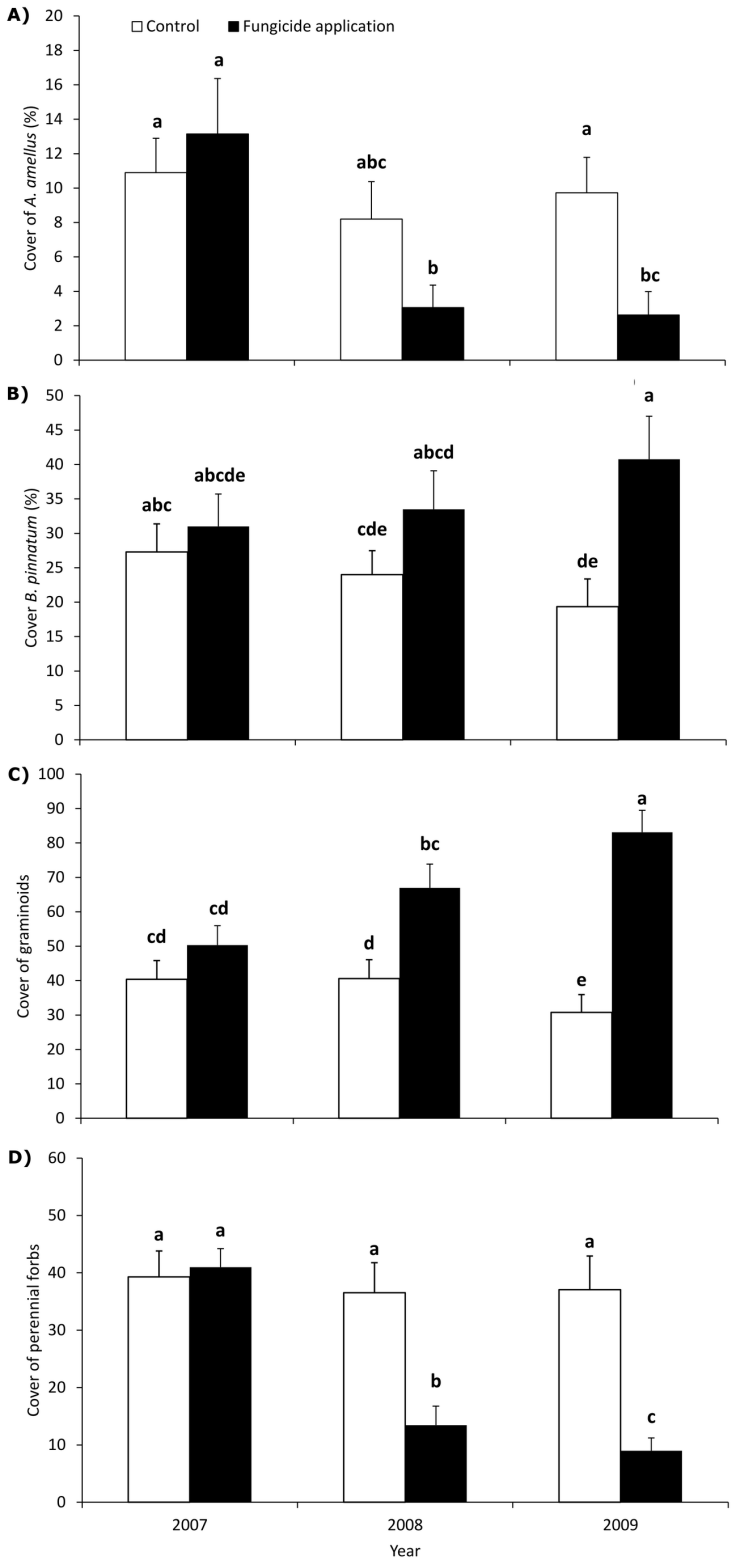
Effect of fungicide treatment on plant growth. Comparison of cover of *Aster amellus* (A), cover of *Brachypodium pinnatum* (B), cover of graminoids (C) and cover of perennial forbs (D) in plots with fungicide application and in control plots during the 3 years of the experiment. The graph shows means and standard errors (n = 10). Columns marked by the same letter are not significantly different (P > 0.05) in a factorial ANOVA. Data from 2007 were collected before fungicide application.

The cover of *A. amellus* significantly decreased with fungicide application (Table 1). Before fungicide application, the mean cover of *A. amellus* was 12 %. At the end of the experiment, the mean cover was 3 % in the plots with fungicide application and 10 % in the control plots (Figure 3A). The cover of dominant *B. pinnatum* significantly increased with fungicide application; the cover in the fungicide-treated plots was almost twice the cover in the control plots at the end of the experiment (Table 1, Figure 3B). The fungicide application also strongly affected the increase in the summed cover of all graminoids (Figure 3C), and the effect was even stronger than the effect on *B. pinnatum*. In contrast, the perennial forbs were strongly suppressed by the fungicide application, as was *A. amellus* (Figure 3D).

**Figure 4:**
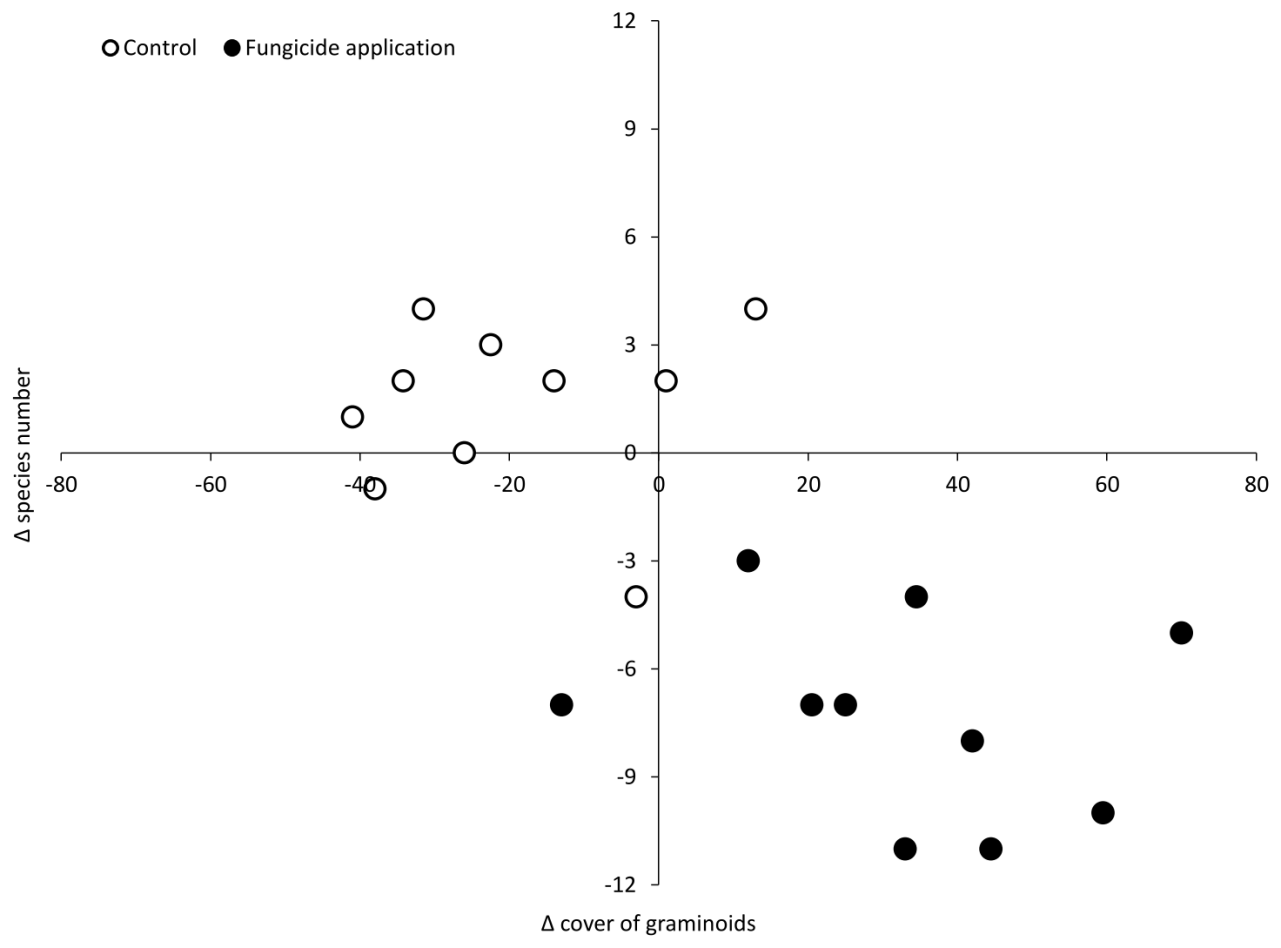
Effect of cover of graminoids on species richness. Significant negative correlation between change in cover of graminoids and change in species richness in plots with and without 3 years of fungicide application (F_1,18_ = 19.51; P < 0.001; linear regression).

In plots with and without fungicide application, there was also a negative relationship between the change in graminoid cover and the change in species richness (F_1,18_ = 19.51; P < 0.001; Figure 4). 

The MIP in the soil sampled in the control plots was twice as high as the MIP in the fungicide-treated plots (9.5 % and 4.1 % of the root length of the host plant colonized by AMF, respectively; F_1,118_ = 23.48; P < 0.001), confirming the successful suppression of AM fungi by fungicide in the experiment.

*B. pinnatum* showed a higher phosphorus concentration in aboveground biomass in the control plots than in the plots where fungicide was applied (746 and 450 mg/kg, respectively; χ^2^_1_ = 10.14; P = 0.002).

The analysis of the soil chemical properties in plots with and without fungicide application showed significant differences only in pH_H20_ (χ^2^_1_ = 14.35; P < 0.001), with higher values of pH in the control plots (Table 2). Despite significant differences in pH_H20_ between the plots with and without fungicide application, the differences in pH_H20_ did not have a significant effect on the plant species composition, number of plant species, cover of *A. amellus*, cover of *B. pinnatum*, cover of graminoids or cover of perennial forbs if fungicide application was used as the covariate (Table 3). However, if we used pH_H20_ as the covariate, the effect of fungicide application on the plant species composition, number of plant species, cover of *A. amellus*, cover of *B. pinnatum*, cover of graminoids and cover of perennial forbs in 2009 still explained a substantial amount of variability in the data (Table 3). There were no significant differences in the concentrations of Mg, Ca, K, P, N, C_tot_, C_(C03)2-_ or C_ox_ between the plots with and without fungicide application (Table 2).

**Table 2:** Chemical characteristics of the soils in the last year of the experiment.

Fungicide application	pH_(H2O)_	pH_(KCl)_	N	C_tot_	C_(C03)2-_	C_ox_		Ca	Mg	K	P
			(%)					(mg kg^-1^)			
No	8.1	7.6	0.2	4.6	2.1	2.5		16633	119	149	4.0
Yes	7.7	7.5	0.2	4.7	2.1	2.6		17172	111	134	5.1
Significance	***	**	n.s.	n.s.	n.s.	n.s.		n.s.	n.s.	n.s.	n.s.

Mean values are shown (n=10). Tests were performed using a Kruskal-Wallis test with fungicide application as an independent variable. ***P ≤ 0.001, **P ≤ 0.01, n.s. P > 0.05.

For Ca, Mg, K and P, available concentrations are given.

**Table 3:** Effects of fungicide application without and with pH as a covariate and effect of pH without and with fungicide as a covariate on plant species composition, number of species, cover of *Aster amellus*, cover of *Brachypodium pinnatum*, cover of graminoids and cover of perennial herbs in the last year of the experiment.

Effect of	Covariate	Resid. df	Plant species composition			Number of plant species			Cover of *A. amellus*			Cover of *B. pinnatum*			Cover of graminoids			Cover of perennial forbs		
			%	F		R^2^	F		R^2^	F		R^2^	F		R^2^	F		R^2^	F	
Fungicide	-	9	26.7	6.6	**	0.77	90.2	***	0.29	5.6	*	0.29	9.9	*	0.67	32.6	***	0.50	18.2	**
Fungicide	pH	8	13.8	2.7	*	0.11	11.1	*	0.29	6.6	*	0.18	5.0	+	0.27	15.9	**	0.11	3.5	+
pH	-	9	17.4	3.8	*	0.66	33.7	***	0.13	2.8	n.s.	0.15	4.3	+	0.45	9.8	*	0.40	10.1	*
pH	Fungicide	8	2.9	0.5	n.s.	0.00	0.0	n.s.	0.12	1.9	n.s.	0.04	1.2	n.s.	0.05	2.8	n.s.	0.00	0.1	n.s.

Plant species composition was tested using a multivariate redundancy analysis, number of plant species using a GLM with a Poisson distribution and cover of *A. amellus*, *B. pinnatum*, graminoids and perennial forbs using a factorial ANOVA.***P ≤ 0.001, **P ≤ 0.01, *P ≤ 0.05, n.s. p > 0.05.

## Discussion

The suppression of AMF using the fungicide carbendazim in the studied dry grassland resulted in substantial changes in plant species composition. Furthermore, there was also a significant decrease in species richness, the cover of the highly mycorrhiza-dependent species *A. amellus* and perennial forbs in the plots where the fungicide was applied. In contrast, we recorded a substantial increase in the cover of the dominant species *B. pinnatum* and graminoids in fungicide-treated plots.

The decrease in species richness in the fungicide-treated plots is in agreement with the theoretical expectations of Hartnett and Wilson [12] as well as with previously published studies [1,15,26]. However, the decrease in species richness reported in this study (a 50 % reduction) is much higher than that found in these previous studies. Gange et al. [1] reported a 25 % reduction in species richness after 3 years. Dhillion and Gardsjord [26] did not record any net fungicide effect on species richness, but they found a 34 % reduction in interaction with grazing after 4 years. Karanika et al. [15] recorded a 21 % reduction in species richness after 2 years of fungicide application. We assume that the decrease in species richness is primarily due to the suppression of mycorrhizal-dependent rare perennial forbs and the expansion of competitively stronger graminoids.

The reason for the stronger effect of fungicide application on AMF in our study might related to the stronger effect of fungicide described for dry habitats [7] or to the absence of dense ground cover, as dense ground cover obstructs the penetration of the soil by the fungicide [56] . 

The greater decrease in species richness in this study than in other studies may also be related to the characteristics of the study site. Our locality is extremely nutrient-poor and dry. For this reason, the plants are much more dependent upon mycorrhizae than they are at other localities. For example, the concentration of available phosphorus in the similar study by Karanika et al. [15] was 3 - 11 mg/kg. In the study of Dhillion and Gardsjord [26], it was even greater, 26 - 67 mg/kg, much greater than the concentration of available phosphorus at our study site (3 - 5 mg/kg). A relationship between lower species richness in fungicide-treated plots and low phosphorus concentration in the studied soils was also proposed by Karanika et al. [15]. These authors stated that despite the general decline of phosphorus concentrations in the species of the fungicide-treated plots, the negative growth effects were observed only in the perennial forbs that had generally higher phosphorus concentrations in their biomass in comparison to graminoids and have therefore greater phosphorus requirements. Thus, the negative effects of fungicide application on phosphorus uptake could decrease the competitive ability of the highly phosphorus-demanding perennial forbs against the graminoids and consequently restrict their growth. This effect may be especially strong in our system, as the soil at the study site is extremely phosphorus poor. As AMF can act as a support systems for seedling establishment [57,58], another explanation for lower species richness in the fungicide-treated plots is that the fungicide reduced the support for seedlings normally furnished by the AMF. This explanation could also be valid in our system, as the clonally growing graminoids are less dependent on generative reproduction than the perennial forbs. 

The species that notably increased their cover in the fungicide-treated plots were the sedge *Carex flacca* and the 2 C3 grasses *Bromus erectus* and *B. pinnatum*. Sedges are well known for their low mycotrophy, e.g., [59,60], and C3 grasses are usually also less mycorrhiza-dependent than most perennial forbs [14,31].

An increased cover of *B. pinnatum* was recorded even though we also found a significantly lower phosphorus concentration in its aboveground biomass in the fungicide-treated plots. In addition, in a study from the same locality (H. Pánková et al., unpublished data), we found that *B. pinnatum* shows 80-90 % root colonization by AMF in the field. Accordingly, it is probable that phosphorus is supplied via mycorrhizal pathway. Nevertheless, it can be expected that *B. pinnatum*, like other C3 grasses, is less mycorrhiza-dependent than the perennial forbs and will, for this reason, most likely increase its dominant position after fungicide application. In the case of *B. pinnatum*, this ability to increase may be related to its superior ability to acquire and store nutrients [61]. The lower concentration of phosphorus in its biomass suggests that *B. pinnatum* is better able to manage acquired phosphorus than the perennial forbs and is thus able to grow efficiently even with a low phosphorus supply. An alternative explanation for the lower phosphorus content in the aboveground biomass of *B. pinnatum* in fungicide-treated plots is the higher dilution of phosphorus in the increased amount of biomass of *B. pinnatum* that appeared when it overdominated small mycorrhiza-dependent species. Although the increase of *B. pinnatum* was not extremely great, as the significant difference between the fungicide-treated and non-treated plots was partly caused by a decrease in the cover of *B. pinnatum* in control plots, the increase in the summed cover of all graminoids was highly significant in the fungicide-treated plots.

The 3 species supported by fungicide application are among the most common species at the study site. *B. pinnatum* is the primary dominant species, and all of the dominant species are graminoids. Accordingly, as predicted by Hartnett and Wilson [12], the fungicide application at the study site produced a significant decrease in species richness. The increased dominance of the previously dominant species further decreased the abundance of the other plant species at the locality [14].

Indeed, among the species most strongly suppressed in the fungicide-treated plots were weak competitors, such as the annual herb *Linum catharticum*, or perennial forbs, such as *A. amellus*, *Potentilla heptaphylla*, *Euphorbia cyparissias* and *Knautia arvensis*. Most of these species were previously characterized as strongly mycorrhiza dependent [47,48,62-64]. In *A. amellus*, Sudová et al. (2010) suggested that the hexaploid cytotype present at the study locality was much less mycorrhiza dependent than the diploid cytotype previously studied. The results of this study, however, suggest that the hexaploid cytotype may also be strongly mycorrhizal dependent under natural conditions. Most of these species also possess little or no clonal growth and are thus, most likely, unable to compete with clonally reproducing grasses and sedges [65,66]. It is also known that not only the presence or absence of AMF but also the diversity and identity of AMF are the determinants of plant diversity and community structure [11]. The fungicide might suppress certain AMF species on which rare plant species are specifically dependent, resulting in the extinction of rare plant species and a decrease in species richness.

Newsham et al. [67] found significant effects of fungicide application on root pathogens and AMF and a significant impact of root pathogenic fungi on plant performance. Thus, another explanation for the increased cover of graminoids could be the negative effect of the fungicide carbendazim on pathogenic fungi in the soil [4] or other components of the soil biota [26], which could result in increased plant growth. The enhanced growth of species less dependent on mycorrhizae (sedges and C3 grasses) could result from the suppression of pathogens. In contrast, the positive effect of release from pathogens on the more mycorrhiza-dependent species (perennial forbs) could be overwhelmed by the negative effects due to suppression of their symbiotic AMF. An increased dominance of graminoids in the community was evident as a result of the changed competitive balance. In contrast, Allison et al. [6], for example, did not find fungicide effects on the soil microbial community in their study.

Differences in plant species composition and number of species between plots with and without fungicide application are occasionally ascribed to increased soil nutrient availability caused by fungicide application [6,68,69]. For example, Allison et al. [6] found increased soil nitrogen content in fungicide-treated plots. They explained this result by citing the observation that the fungicide benomyl contains 19.3 % of nitrogen [70]. However, this effect of fungicide application is usually observed only if a high dosage of fungicide is applied. Such effects of benomyl, or the functionally identical fungicide carbendazim used in this study, are usually very small [69]. The only significant difference in soil chemical composition between plots with fungicide addition and the controls in our study was a slightly more acidic pH in the plots with fungicide application; no significant changes in nutrient concentrations were observed. Moreover, the effect of fungicide application on plant species composition and species richness was evident even after using pH as a covariate in the models. 

## Conclusions

The suppression of AMF using the fungicide carbendazim in the studied dry grassland resulted in substantial changes in plant species composition and a significant decrease in species richness. The only species to benefit from the fungicide application were graminoids - 2 dominant C3 grasses, *B. pinnatum* and *B. erectus*, and the sedge *Carex flacca*. These species appear to be less mycorrhiza dependent and are, most likely, able to compete effectively with more mycorrhiza-dependent perennial forbs due to their clonal growth and efficient nutrient usage. Our results thus suggest that AMF are an essential part of the soil communities supporting a high diversity of plant species in species-rich dry grasslands at nutrient-poor habitats. AMF are particularly important for the maintenance of the populations of perennial forbs, many of which are rare and endangered in the area. The high sensitivity of the system to the removal of AMF also suggests that the system is very sensitive to external interventions. Accordingly, it can be expected that the recovery of the plant communities after an external disturbance (e.g., tillage) that tends to disrupt of the AMF community will be extremely slow. 

## References

[1] Gange AC, Brown VK, Sinclair GS (1993) Vesicular-Arbuscular Mycorrhizal Fungi: A Determinant of Plant Community Structure in Early Succession. Funct Ecol 7: 616-622. doi:10.2307/2390139.

[2] Francis R, Read D (1994) The contributions of mycorrhizal fungi to the determination of plant community structure. Plant Soil 159: 11-25.

[3] Smith MD, Hartnett DC, Wilson GWT (1999) Interacting influence of mycorrhizal symbiosis and competition on plant diversity in tallgrass prairie. Oecologia 121: 574-582. doi:10.1007/s004420050964. 28308367

[4] Schweiger P, Spliid N, Jakobsen I (2001) Fungicide application and phosphorus uptake by hyphae of arbuscular mycorrhizal fungi into field-grown peas. Soil Biol Biochem 33: 1231-1237. doi:10.1016/S0038-0717(01)00028-1.

[5] Smith SE, Read DJ (2008) Mycorrhizal Symbiosis. Academic Press. 816 pp.

[6] Allison V, Rajaniemi T, Goldberg D, Zak D (2007) Quantifying direct and indirect effects of fungicide on an old-field plant community: an experimental null-community approach. Plant Ecol 190: 53-69. doi:10.1007/s11258-006-9190-8.

[7] Šmilauer P, Šmilauerová M (2000) Effect of AM Symbiosis Exclusion on Grassland Community Composition. Folia Geobot 35: 13-25. doi:10.1007/BF02803084.

[8] Hartnett DC, Hetrick BAD, Wilson GWT, Gibson DJ (1993) Mycorrhizal Influence on Intra- and Interspecific Neighbour Interactions among Co-Occurring Prairie Grasses. J Ecol 81: 787-795. doi:10.2307/2261676.

[9] Hetrick B, Hartnett D, Wilson G, Gibson D (1994) Effects of Mycorrhizae, Phosphorus Availability, and Plant-Density on Yield Relationships Among Competing Tallgrass Prairie Grasses. Can J Bot 72: 168-176. doi:10.1139/b94-023.

[10] Van der Heijden MGA, Boller T, Wiemken A, Sanders IR (1998) Different Arbuscular Mycorrhizal Fungal Species Are Potential Determinants of Plant Community Structure. Ecology 79: 2082-2091. doi:10.1890/0012-9658(1998)079[2082:DAMFSA]2.0.CO;2.

[11] Van der Heijden MGA, Klironomos JN, Ursic M, Moutoglis P, Streitwolf-Engel R et al. (1998) Mycorrhizal fungal diversity determines plant biodiversity, ecosystem variability and productivity. Nature 396: 69-72. doi:10.1038/23932.

[12] Hartnett DC, Wilson GWT (1999) Mycorrhizae Influence Plant Community Structure and Diversity in Tallgrass Prairie. Ecology 80: 1187-1195. doi:10.1890/0012-9658(1999)080[1187:MIPCSA]2.0.CO;2.

[13] O’Connor PJ, Smith SE, Smith FA (2002) Arbuscular mycorrhizas influence plant diversity and community structure in a semiarid herbland. New Phytol 154: 209-218. doi:10.1046/j.1469-8137.2002.00364.x.

[14] Grime J, Mackey J, Hillier S, Read D (1987) Floristic Diversity in a Model System Using Experimental Microcosms. Nature 328: 420-422. doi:10.1038/328420a0.

[15] Karanika E, Mamolos A, Alifragis D, Kalburtji K, Veresoglou D (2008) Arbuscular mycorrhizas contribution to nutrition, productivity, structure and diversity of plant community in mountainous herbaceous grassland of northern Greece. Plant Ecol 199: 225-234. doi:10.1007/s11258-008-9427-9.

[16] Fitter A (1986) Effect of Benomyl on Leaf Phosphorus Concentration in Alpine Grasslands - a Test of Mycorrhizal Benefit. New Phytol 103: 767-776. doi:10.1111/j.1469-8137.1986.tb00851.x.

[17] Fitter AH, Nichols R (1988) The use of benomyl to control infection by vesicular–arbuscular mycorrhizal fungi. New Phytol 110: 201-206. doi:10.1111/j.1469-8137.1988.tb00253.x.

[18] Wilson G, Hartnett D (1997) Effects of mycorrhizae on plant growth and dynamics in experimental tall grass prairie microcosms. Am J Bot 84: 478-478. doi:10.2307/2446024. PubMed: 21708601. 21708601

[19] Wilson GWT, Hartnett DC, Smith MD, Kobbeman K (2001) Effects of mycorrhizae on growth and demography of tallgrass prairie forbs. Am J Bot 88: 1452-1457. doi:10.2307/3558453. PubMed: 21669678. 21669678

[20] McCain K, Wilson G, Blair J (2011) Mycorrhizal suppression alters plant productivity and forb establishment in a grass-dominated prairie restoration. Plant Ecol 212: 1675-1685. doi:10.1007/s11258-011-9940-0.

[21] Helgason T, Merryweather JW, Young JPW, Fitter AH (2007) Specificity and resilience in the arbuscular mycorrhizal fungi of a natural woodland community. J Ecol 95: 623-630. doi:10.1111/j.1365-2745.2007.01239.x.

[22] Zobel M, Pilt I, Moora M, Pärtel M, Liira J (1999) Small-scale dynamics of plant communities in an experimentally polluted and fungicide-treated subarctic birch-pine forest. Acta Oecol 20: 29-37. doi:10.1016/S1146-609X(99)80013-7.

[23] Koorem K, Saks Ü, Sõber V, Uibopuu A, Öpik M et al. (2012) Effects of arbuscular mycorrhiza on community composition and seedling recruitment in temperate forest understory. Basic Appl Ecol 13: 663-672.

[24] Newsham KK, Watkinson AR, West HM, Fitter AH (1995) Symbiotic Fungi Determine Plant Community Structure: Changes in a Lichen- Rich Community Induced by Fungicide Application. Funct Ecol 9: 442-447. doi:10.2307/2390007.

[25] Jordan N, Huerd S (2008) Effects of soil fungi on weed communities in a corn–soybean rotation. Renew Agr Foods Syst 23: 108-117.

[26] Dhillion SS, Gardsjord TL (2004) Arbuscular mycorrhizas influence plant diversity, productivity, and nutrients in boreal grasslands. Can J Bot 82: 104-114. doi:10.1139/b03-139.

[27] Koide RT, Huenneke LF, Hamburg SP, Mooney HA (1988) Effects of Applications of Fungicide, Phosphorus and Nitrogen on the Structure and Productivity of an Annual Serpentine Plant Community. Funct Ecol 2: 335-344. doi:10.2307/2389406.

[28] Blanke V, Wagner M, Renker C, Lippert H, Michulitz M et al. (2011) Arbuscular mycorrhizas in phosphate-polluted soil: interrelations between root colonization and nitrogen. Plant Soil 343: 379-392. doi:10.1007/s11104-011-0727-9.

[29] Bolliger J, Edwards TC Jr., Eggenberg S, Ismail S, Seidl I et al. (2011) Balancing Forest-Regeneration Probabilities and Maintenance Costs in Dry Grasslands of High Conservation Priority. Conserv Biol 25: 567-576. doi:10.1111/j.1523-1739.2010.01630.x. PubMed: 21175843. 21175843

[30] O’Connor P, Manjarrez M, Smith SE (2009) The fate and efficacy of benomyl applied to field soils to suppress activity of arbuscular mycorrhizal fungi. Can J Microbiol 55: 901-904. doi:10.1139/W09-035. PubMed: 19767864. 19767864

[31] Wilson GWT, Hartnett DC (1998) Interspecific variation in plant responses to mycorrhizal colonization in tallgrass prairie. Am J Bot 85: 1732-1738. doi:10.2307/2446507. PubMed: 21680333. 21680333

[32] Gange A, Brown V, Farmer L (1990) A Test of Mycorrhizal Benefit in an Early Successional Plant Community. New Phytol 115: 85-91. doi:10.1111/j.1469-8137.1990.tb00925.x.

[33] Chýlová T, Münzbergová Z (2008) Past land use co-determines the present distribution of dry grassland plant species. Preslia 80: 183-198.

[34] Studnička M (1972) Dry grasslands in České Středohoří : A study of ecology and fytocenology. M.S. thesis Prague: Charles University.

[35] Ellenberg H (1988) Vegetation Ecology of Central Europe. UK: Cambridge University Press. 758 pp.

[36] Raabová J, Münzbergová Z, Fischer M (2007) Ecological rather than geographic or genetic distance affects local adaptation of the rare perennial herb, Aster Amellus. Biol Conserv 139: 348-357. doi:10.1016/j.biocon.2007.07.007.

[37] Münzbergová Z (2004) Effect of spatial scale on factors limiting species distributions in dry grassland fragments. J Ecol 92: 854-867. doi:10.1111/j.0022-0477.2004.00919.x.

[38] Tremlová K, Münzbergová Z (2007) Importance of species traits for species distribution in fragmented landscapes. Ecology 88: 965-977. doi:10.1890/06-0924. PubMed: 17536712. 17536712

[39] Knappová J, Hemrová L, Münzbergová Z (2012) Colonization of central European abandoned fields by dry grassland species depends on the species richness of the source habitats: a new approach for measuring habitat isolation. Landsc Ecol 27: 97-108. doi:10.1007/s10980-011-9680-5.

[40] Knappová J, Knapp M, Münzbergová Z (2013) Spatio-Temporal Variation in Contrasting Effects of Resident Vegetation on Establishment, Growth and Reproduction of Dry Grassland Plants: Implications for Seed Addition Experiments. PLOS ONE 8: e65879. doi:10.1371/journal.pone.0065879. PubMed: 23755288. 23755288 PMC3673946

[41] Environmental Protection Agency (2001) Benomyl; Cancellation Order. Available: http://www.epa.gov/EPA-PEST/2001/August/Day-08/p19572.htm. Accessed 2013 October 17.

[42] Helweg A (1973) Undersoegelser over fungicidet benomyl i jord. I. Stabilitet og biologisk nedbrydning. (Investigations on the fungicide benomyl in soil. I stabilim and biological decomposing.) Statens Forsogs Plant: 232-243..

[43] Tang CS, Yanagihara K, Zhang Y (1992) 1-butyl isocyanate from aqueous Benlate® formulations. Arch Environ Contam Toxicol 23: 270-272. doi:10.1007/BF00212286.

[44] Kahiluoto H, Vestberg M (2000) Creation of a non-mycorrhizal control for a bioassay of AM effectiveness. Mycorrhiza 9: 259-270. doi:10.1007/PL00009990.

[45] Westhoff V, van der Maarel E (1978) The Braun-Blanquet approach. In: Whittaker RH Classification of plant communities. Den Haag, NL: Dr. W Jung. pp. 287-399.

[46] Castro S, Loureiro J, Procházka T, Münzbergová Z (2012) Cytotype distribution at a diploid–hexaploid contact zone in Aster Amellus (Asteraceae). Ann Bot 110: 1047-1055. doi:10.1093/aob/mcs177. PubMed: 22887024. 22887024 PMC3448430

[47] Pánková H, Münzbergová Z, Rydlová J, Vosátka M (2008) Differences in AM fungal root colonization between populations of perennial Aster species have genetic reasons. Oecologia 157: 211-220. doi:10.1007/s00442-008-1064-4. PubMed: 18523810. 18523810

[48] Pánková H, Münzbergová Z, Rydlová J, Vosátka M (2011) The response of Aster Amellus (Asteraceae) to mycorrhiza depends on the origins of both the soil and the fungi. Am J Bot 98: 850-858. doi:10.3732/ajb.0900350. PubMed: 21613062. 21613062

[49] Sudová R, Rydlová J, Münzbergová Z, Suda J (2010) Ploidy-specific interactions of three host plants with arbuscular mycorrhizal fungi: Does genome copy number matter? Am J Bot 97: 1798-1807. doi:10.3732/ajb.1000114. PubMed: 21616819. 21616819

[50] Wilson J, Trinick M (1983) Factors Affecting the Estimation of Numbers of Infective Propagules of Vesicular Arbuscular Mycorrhizal Fungi by the Most Probable Number Method. Aust J Crops Sci 21: 73-81.

[51] Koske R, Gemma J (1989) A Modified Procedure for Staining Roots to Detect VA-Mycorrhizas. Mycol Res 92: 486-505. doi:10.1016/S0953-7562(89)80195-9.

[52] Giovannetti M, Mosse B (1980) An Evaluation of Techniques for Measuring Vesicular Arbuscular Mycorrhizal Infection in Roots. New Phytol 84: 489-500. doi:10.1111/j.1469-8137.1980.tb04556.x.

[53] Ehrenberger F, Gorbach S (1973) Methoden der organischen Elementar- und Spurenanalyse. Weinheim: Verlag Chemie.

[54] Lepš J, Šmilauer P (2003) Multivariate analysis of ecological data using CANOCO. Cambridge University Press. 292 pp.

[55] Ter Braak CJF, Šmilauer P (2002) CANOCO reference manual and CanoDraw for Windows user’s guide: software for canonical community ordination (version 4.5). Microcomputer Power Ithaca, NY.

[56] Pedersen CT, Sylvia DM (1997) Limitations to using benomyl in evaluating mycorrhizal functioning. Biol Fertil Soils 25: 163-168.

[57] Van der Heijden MGA (2004) Arbuscular mycorrhizal fungi as support systems for seedling establishment in grassland. Ecol Lett 7: 293-303. doi:10.1111/j.1461-0248.2004.00577.x.

[58] Jones MD, Smith SE (2004) Exploring functional definitions of mycorrhizas: Are mycorrhizas always mutualisms? Can J Bot 82: 1089-1109. doi:10.1139/b04-110.

[59] Harley JL, Harley EL (1987) A Check-List of Mycorrhiza in the British Flora. New Phytol 105: 1-102. doi:10.1111/j.1469-8137.1987.tb00107.x.

[60] Muthukumar T, Udaiyan K, Shanmughavel P (2004) Mycorrhiza in sedges–an overview. Mycorrhiza 14: 65-77. doi:10.1007/s00572-004-0296-3. PubMed: 14999550. 14999550

[61] Bobbink R (1991) Effects of Nutrient Enrichment in Dutch Chalk Grassland. J Appl Ecol 28: 28-41. doi:10.2307/2404111.

[62] Turnau K (1998) Heavy metal content and localization in mycorrhizal Euphorbia cyparissias zinc wastes in southern Poland. Acta Soc Bot Pol Pol Tow Bot 67: 105-113.

[63] Pendleton RL, Pendleton BK, Howard GL, Warren SD (2004) Response of Lewis flax seedlings to inoculation with arbuscular mycorrhizal fungi and cyanobacteria. In: Hild AL Shaw NL Meyer SE Seed and soil dynamics in shrubland ecosystems. USDA Forest Service Proceedings. Available: http://www.fs.fed.us/rm/pubs/rmrs_p031/rmrs_p031_064_068.pdf. Accessed 2013 February 26.

[64] Doubková P, Suda J, Sudová R (2011) Arbuscular mycorrhizal symbiosis on serpentine soils: the effect of native fungal communities on different Knautia arvensis ecotypes. Plant Soil 345: 325-338. doi:10.1007/s11104-011-0785-z.

[65] Grime JP, Hodgson JG, Hunt R (1988) Comparative plant ecology. A Funct Approach Common British Species: 742.

[66] Crawley M (2009) Plant Ecology. John Wiley & Sons. 742 pp.

[67] Newsham KK, Fitter AH, Watkinson AR (1994) Root Pathogenic and Arbuscular Mycorrhizal Fungi Determine Fecundity of Asymptomatic Plants in the Field. J Ecol 82: 805-814. doi:10.2307/2261445.

[68] Burrows LA, Edwards CA (2000) The effects of the fungicide carbendazim in an innovative integrated terrestrial microcosm system. British Crop Protection Council pp. 365-370.

[69] Chen S-K, Edwards CA (2001) A microcosm approach to assess the effects of fungicides on soil ecological processes and plant growth: comparisons of two soil types. Soil Biol Biochem 33: 1981-1991. doi:10.1016/S0038-0717(01)00132-8.

[70] Mazur AR (1977) Soil microorganisms and preventive fungicide programs - What are the interactions?. Available: http://gsr.lib.msu.edu/1970s/1977/770504.pdf. Accessed 2013 October 17.

